# Biopolymeric Hydrolysates from *Dosidicus gigas*: Functional Applications and Shelf-Life Extension in Squid Sausages

**DOI:** 10.3390/polym17070839

**Published:** 2025-03-21

**Authors:** Francisco Antonio López-Medina, Octavio Dublán-García, Ana Gabriela Morachis-Valdez, Karinne Saucedo-Vence, Guadalupe López-García, Daniel Díaz-Bandera, Rosa María Gómez-Espinoza

**Affiliations:** 1 Laboratorio de Alimentos, Facultad de Química, Unidad Cerrillo, Universidad Autónoma del Estado de México, Carretera Toluca—Ixtlahuaca Kilómetro 15.5, El Cerrillo Piedras Blancas, Toluca C. P. 50200, Mexico; flopezm@uaemex.mx (F.A.L.-M.); c250028@iuem.edu.mx (G.L.-G.); ddiazb@uaemex.mx (D.D.-B.); rmgomeze@uaemex.mx (R.M.G.-E.); 2 Unidad Académica de Capulhuac, Universidad Tecnológica del Valle de Toluca (UTVT), Calle s/n, 611 Oriente de, Colonia, Lomas de San Juan Municipio, Capulhuac de Mirafuentes C. P. 52700, Mexico; karinne.saucedo@utvtol.edu.mx

**Keywords:** protein hydrolysates, jumbo squid, *Bacillus subtilis*, antioxidant activity, enzymatic inhibition, functional foods, prolongate storage stability, marine biopolymers

## Abstract

Bioactive protein hydrolysates from Dosidicus gigas, obtained via Bacillus subtilis fermentation (20 °C, 4–8 h), were assessed for functional properties and their impact on jumbo squid sausage preservation. The hydrolysates exhibited strong antioxidant activity (742.17 μmol TE/g) and inhibited key metabolic enzymes: α-glucosidase (93.29%), α-amylase (20.87%), lipase (35.44%), and ACE-I (88.96%), indicating potential benefits for managing diabetes, obesity, and hypertension. Sausages enriched with 0.1% hydrolysates, stored at 4 °C, had a 95.5% longer shelf life (43 vs. 22 days), reduced microbial spoilage (TVC: 3.68 vs. 5.42 Log CFU/g), and 35.6% lower total volatile bases. Water-holding capacity improved (88.21% vs. 87.15%), and oxidative browning was delayed, preserving color stability. These results highlight D. gigas hydrolysates as multifunctional bioactive compounds with potential as natural stabilizers in clean-label formulations. Their capacity to enhance food stability and replace synthetic preservatives offers a sustainable, innovative strategy for the functional food industry.

## 1. Introduction

Functional foods have gained significant attention due to their dual role in providing essential nutrients and promoting health [1]. This growing interest is driven by increased consumer awareness of chronic diseases such as diabetes, obesity, and hypertension, which are prevalent worldwide [2]. The demand for food products that offer health benefits has spurred research into bioactive compounds, among which protein hydrolysates derived from marine resources stand out for their antioxidant properties, enzyme inhibition capacity, and lipid metabolism modulation [3,4,5,6].

In this context, sustainability in the food industry has become a key factor, promoting the reuse of marine by-products for the formulation of functional ingredients over land sources, specifically due to the presence of omega 3 and the amino-acid profile [7,8]. *Dosidicus gigas* represents an abundant biomass source with high protein content, whose valorization can significantly contribute to the circular economy in aquaculture [7,9]. Up to 75% of the total weight of this cephalopod is discarded as waste, including skin, head, and viscera, even though these by-products contain bioactive compounds with technological and nutritional potential [10].

To place this study within the context of biopolymer science, it is essential to recognize that protein hydrolysates from *Dosidicus gigas* can be considered biopolymers due to their peptide-based structure and amphiphilic properties [11,12]. The presence of functional groups such as hydroxyl (-OH), carbonyl (C=O), and aromatic rings influences their bioactivity and structural functionality. These characteristics affect key technological properties such as emulsification, water-holding capacity, and oxidative stability, which are crucial in the food industry [13,14].

FTIR analysis of these hydrolysates confirms their structural complexity, revealing functional groups associated with antioxidant and antimicrobial activities. These properties enable their potential use as natural stabilizers in foods, reducing dependency on synthetic polymers and artificial preservatives in response to the growing demand for clean-label products [15,16,17,18,19]. Additionally, the amphiphilic nature of hydrolysates enhances their integration into emulsion systems, improving stability and preventing phase separation [16].

Recent studies have demonstrated that protein hydrolysates can enhance the stability of meat products by reducing lipid oxidation and improving water retention [10,15]. However, their application in highly perishable food matrices remains an underexplored area [20]. Squid sausages, due to their high water content and proteolytic activity, pose challenges in stability and shelf life [21,22], making them an ideal model for evaluating the effectiveness of bioactive hydrolysates in improving food stability [23].

This study focuses on incorporating bioactive protein hydrolysates from *Dosidicus gigas* into jumbo squid sausages, enriched with inulin and orange fiber. The hydrolysates, produced by *Bacillus subtilis* fermentation, enhance protein bioactivity and contribute to food sustainability. The research provides a practical approach to improving the stability, functionality, and nutritional value of squid sausages while utilizing marine resources efficiently.

## 2. Materials and Methods

### 2.1. Samples and Reagents

*Dosidicus gigas* frozen mantle was purchased from a local market 15 days post-capture. The samples were transported in an insulated container with dry ice to maintain a temperature of −20 °C. Upon arrival at the laboratory, the samples were stored in a freezer at −20 °C until further processing. Prior to use, the mantle was thawed overnight at 4 °C under controlled conditions to minimize quality degradation. *Bacillus subtilis* (ATCC 6633) was purchased from Thermo Fischer Scientific (Lenexa, KS, USA), and 2,2′-azobis(2-methylpropionamidine) dihydrochloride (AAPH), Trolox, α-glucosidase from *Saccharomyces cerevisiae*, p-nitrophenyl-α-glucopyranoside (pNPG), acarbose, α-amylase from *Bacillus licheniformis*, starch, dinitrosalicylic acid, ρ-nitrophenyl palmitate (ρNPP), porcine pancreatic lipase type II (L-3126), Orlistat, N-hippuryl-histidil-leucine, rabbit lung angiotensin-converting enzyme, Captopril, NaOH, HCl, methyl red, methylene blue, and inorganic salts used to prepare buffers were purchased from Sigma-Aldrich (St. Louis, MO, USA). Peptone, plate count agar, and Violet Red Bile Agar were purchased from Difco™ BD-VWR (Sparks, MD, USA). All other materials used were of food grade.

### 2.2. Production of Protein Hydrolysates

Based on previous studies [11], the conditions were chosen to obtain five hydrolysates with antioxidant and antimicrobial properties. Each fermentation condition was performed in triplicate to ensure reproducibility. For each batch, three independent samples were prepared and analyzed for all experimental parameters, using *Bacillus subtilis* (ATCC 6633) to ferment culture media with muscle and collagen from giant squid, as a nitrogen source. The chosen conditions were as follows: (H1) 4 h of fermentation, 25% collagen, 75% muscle; (H2) 4 h of fermentation, 100% collagen; (H3) 8 h of fermentation, 100% muscle; (H4) 8 h of fermentation, 50% collagen, 50% muscle; and (H5) 8 h of fermentation, 100% collagen. In addition to these, a non-fermented control (H_0_) consisting of unprocessed muscle and collagen was included to assess baseline properties. A standardized fermentation condition (SFC) was used as a positive control, following the protocol described by Marti-Quijal et al. (2020) [19]. The culture medium was prepared by dissolving 30 g of a nitrogen source, consisting of a mixture of collagen and mantle powder, in 1 L of phosphate–citrate–bicarbonate buffer (50 mM–150 mM–150 mM, pH 7.5) with NaCl (200 mM). The solution was then sterilized by autoclaving at 121 °C for 15 min.

The *Bacillus subtilis* ATCC 6633 strain was initially cultured on nutrient agar at 20 °C for 22 h. Subsequently, it was inoculated into a nutrient broth and incubated at 20 °C for 18 h under constant agitation at 120 rpm. The final inoculum concentration was determined to be 4 × 10⁶ CFU/mL.

The inoculation was performed by adding 40 mL of the inoculum to 1 L of the previously sterilized culture medium. Fermentation was carried out at 20 °C under agitation (120 rpm) for 4 and 8 h. The fermentation products were then separated by centrifugation at 8500× *g* for 30 min at 4 °C.

For subsequent use, precipitation was carried out using 96° ethanol at a 1:1 ratio with the hydrolyzed liquid. The precipitated fractions were separated by decantation and dried at 30 °C for 72 h. Finally, the obtained fractions were subjected to a lyophilization process. Previous studies [11] characterized the fermentation products of *Bacillus subtilis*, identifying bioactive compounds of interest. In the present study, the oily phase obtained after fermentation and subsequent ethanol precipitation was utilized, as it concentrates secondary metabolites associated with the fermentation process.

To ensure the reliability and reproducibility of the analytical results, all methods were validated according to standard protocols. Validation parameters included Limit of Detection (LOD) and Limit of Quantification (LOQ), and UV–visible spectrophotometer (Científica Vela Quin, Mexico City, Mexico, Model VE 5600UV) specifications are as follows: wavelength accuracy: ±0.5 nm, wavelength repeatability: 0.2 nm, photometric accuracy: ±0.3% T, photometric repeatability: ±0.2% T, stability: 0.002 A/h at 500 nm, and stray light: ≤0.05% T at 220 nm and 360 nm.

### 2.3. Characterization of the Protein Hydrolysates

The hydrolyzed samples were diluted to a concentration of 40 mg/mL for the determination of antioxidant activity and enzyme inhibition.

#### 2.3.1. Oxygen Radical Absorbance Capacity (ORAC)

The ORAC assay was carried out using the method reported by Sae-leaw et al. [24], with slight modifications, using 2,2′-azobis(2-methylpropionamidine) dihydrochloride (AAPH) as a peroxyl radical, and fluorescein as the fluorescent probe. The loss of fluorescence was measured every 70 s for 70 min, with a wavelength of 485 nm for excitation and 535 nm for emission, using a spectrophotometer. Results were calculated using a Trolox standard curve and expressed as µmol of Trolox equivalents per gram of sample (µmol TE/g sample). The calibration curve was constructed at concentrations ranging from 6.25 to 125 µM. The regression equation obtained was y = 42.1x + 937, with a coefficient of determination (R^2^ = 0.9987), indicating high linearity. Furthermore, the method validation included the following parameters: the LOD and LOQ were 0.02 µmol TE/mL and 0.1 µmol TE/mL, respectively.

#### 2.3.2. Enzymatic Inhibition

The percentage of inhibition was calculated using the following equation:

Inhibition % = (Abs blank − Abs sample) Abs blank × 100 (1)

where Abs blank is the absorbance of the control (blank, without sample), and Abs sample is the absorbance in the presence of the sample. For enzymatic inhibition assays, the LOD and LOQ were determined as 0.01 µmol inhibitor/mL and 0.05 µmol inhibitor/mL, respectively. These validation steps ensured that the data obtained were robust and consistent across replicates.

##### Enzymatic Inhibition of α-Glucosidase

α-glucosidase inhibitory activity was determined via a modified assay from the Worthington Enzyme Manual, as reported by Picos-Salas et al. [25]. Enzyme activity was determined by measuring the release of p-nitrophenol from the p-nitrophenyl-α-glucopyranoside (pNPG) substrate by α-glucosidase from *Saccharomyces cerevisiae* (0.6 U/mL). Absorbance at 405 nm was measured with a 96-well microplate reader. Also, 50 µL of 1 mM acarbose was used as a control.

##### Inhibition of α-Amylase

α-amylase inhibitory activity was determined via a modified assay from the Worthington Enzyme Manual, as reported by Picos-Salas et al. [25]. The reaction was performed at 37 °C for 10 min with 50 µL of pancreatic α-amylase at 13 U/mL, 50 µL of starch at 1%, and 50 µL of sample. Afterwards, 1 mL of dinitrosalicylic acid reagent was added, and the solution was heated at 85 °C for 15 min. Then, the solution was transferred to ice to cool down to room temperature (20 ± 2 °C), and 1 mL of distilled water was added. Finally, 250 µL from each sample was placed in a 96-well plate, and the absorbance was recorded at 540 nm using a microplate reader. Fifty microliters of 1 mM acarbose was used as a control.

##### Inhibition of Lipase

The lipase inhibitory activity was determined using a spectroscopic method developed by Vo et al. [26]. In the assay, ρ-nitrophenyl palmitate (ρNPP) was used as substrate, which is hydrolyzed by lipase to ρ-nitrophenol (ρNP). The released ρNP was monitored at 410 nm for 10 min (at 37 °C) using a microplate reader. Orlistat^®^ (1 mg/mL, AMSA Laboratories, Coyoacán, Mexico City, Mexico) was used as a reference inhibitor.

##### Angiotensin-Converting Enzyme Inhibition Assay (ACE)

ACE inhibitory activity of the extracts was determined using the method developed by Korczek et al. [27], with some modifications. Briefly, 50 μL of sample was dissolved in 200 μL of 0.1 M borate buffer containing 0.3 M NaCl (pH 8.3) and mixed with 50 μL of ACE solution (2 mU/mL). The mixture was then pre-incubated at 37 °C for 10 min; after this time, 150 μL of 5.0 mM substrate (hippuryl-histidyl-leucine) was added. Liberation of hippuric acid was determined by absorbance, using a photodiode array spectrophotometer (Synergy HT, BioTek Instruments, Inc., Winooski, VT, USA) at 288 nm, dispensing accuracy ±1 μL, precision ±2%. Blank samples were prepared without addition of enzyme, and control samples were prepared without the addition of hydrolysates. Captopril (1.0 mg/mL in final assay mixture) was used as a reference inhibitor.

#### 2.3.3. Infrared Spectroscopy

According to Djellouli et al. [13], the infrared spectra of the lyophilized hydrolysates were obtained using 1 mg of sample at room temperature (20 + 2 °C), with a Jasco FT/IR 400 spectrometer (Jasco, Tokyo, Japan) at a range of 600–4000 cm^−1^, with a resolution of 4 cm^−1^, wavelength accuracy: ±0.01 cm^−1^, transmission accuracy: ±0.1% T, wavelength repeatability: 0.005 cm^−1^, transmission repeatability: ±0.05% T, Signal-to-Noise Ratio (SNR): >30,000:1, and detection limit: 0.01% T.

### 2.4. Development of the Giant Squid Sausage

Frozen jumbo squid mantle was used for 14 days post-capture, separating the inner and outer layers of collagen from the muscle before use. The percentage used of each ingredient was determined by the research group through previous studies [28]. Ingredients were added to the cutter in the following order: jumbo squid muscle, sodium chloride, phosphate blend, water/ice, oil, spices, maltodextrin, starch, and fiber blend. The formulation used was the following (*w*/*w* percentage): jumbo squid muscle (70.5%), water/ice (3:7) (10%), canola oil (6%), citrus fiber blend (CITRI-FI™ (Fibers Inc., Willmar, MN, USA) 100)/inulin (5:5) (4%), maltodextrin (100 DE) (4%), corn starch (2%), mixed phosphates (sodium monobasic 23 g: sodium dibasic 47 g) (1.4%), spices (cloves, cumin, cinnamon, walnut nutmeg, basil, dill, coriander, garlic, black pepper, ginger, turmeric, chipotle chili in adobo—equivalent to 1% *w*/*w* in the final formulation) (1.4%), and sodium chloride (0.7%).

### 2.5. Physicochemical Properties of the Sausage

#### 2.5.1. Proximal Analysis and Microbiological Quality

Determined by AOAC methods [29]: moisture, protein, fat, fiber, and ash of jumbo squid sausages, as well as “carbohydrate by difference”, in addition to the presence of *S. aureus*, *Salmonella* spp., and Coliforms.

#### 2.5.2. Physicochemical Properties of Sausages During Storage

##### Determination of pH

According to what was described by Verma et al. [30], 10 g of sample was homogenized with 90 mL of distilled water for 1 min, with an Osterizer blender, and the resulting solution was filtered with Whatman^®^ quantitative filter paper (ashless, Grade 42), (Cytiva, Maidstone, UK). The pH was determined using a potentiometer (Conductronic PH 120; Mexico City, Mexico).

##### Determination of Total Volatile Bases (TVB)

TVB content was determined using the Conway microdiffusion method, according to Riebroy et al. [31]. For the titration, the Conway indicator (0.125 g of methyl red in 10 mL of 0.05 N NaOH, 0.080 g of methylene blue in 90 mL of distilled water) and a 0.01 N HCl solution were used until the green color changed to pink. Results were expressed as mg N/100 g of sausage.

##### Determination of Water-Holding Capacity (WHC)

It was determined according to the method proposed by Zhou et al., with modifications. Polycarbonate cups, with a capacity of 250 mL and previously weighed, were filled with 2 circles of filter paper and 5 g of sample [32]. Afterwards, they were centrifuged at 3000× *g* for 60 min at 4 °C. The water-holding capacity was calculated using the following formula:

WHC % = (weight after centrifugation/initial weight) × 100

##### Color

Color of the sausages was determined using a Chroma Meter (Minolta Camera Co., Ltd., CR-200, Osaka, Japan) with the CIE L* a* b* color system. The instrument was standardized via a black-and-white Minolta calibration plate. The values were shown as L* (i.e., lightness), a* (i.e., redness), and b* (i.e., yellowness) [32].

##### Firmness Analysis

The sausages were portioned into standard measurements with a height of 25 mm. Hardness testing was performed using a TA-XT2 (EXPONENT_5.1.1.0) texture analyzer. A cylindrical probe with a 10 mm diameter was used, attached to a 25 kg load cell. The compression height was set to 10 mm, with a deformation speed of 1 mm/s [32].

##### Microbiological Analysis

The plate count method was used; 10 g of sample were homogenized with 90 mL of 0.1% peptone solution. Decimal dilutions were made from this dilution, placing it on a plate with the addition of plate count agar. For the determination of the total viable count (TVC), the inoculated plates were incubated at 35 °C for 48 h and for 7 days at 4 °C to determine the psychrophilic bacteria count (PBC). To determine total coliforms, Violet Red Bile Agar (VRBA) was added to the inoculated plates, incubating them at 36 °C for 24 h. Counts were expressed as logarithms of colony-forming units per gram (Log CFU/g), and were carried out in duplicate [32,33,34].

### 2.6. Statistical Analysis

Tests were performed in quintuplicate. Data were analyzed using Statgraphics Centurion^®^ v. 16.103, using Tukey’s post hoc test with a confidence level of *p* < 0.05. All graphical representations of data, including bar plots, scatter plots, and trend analyses, were generated using GraphPad Prism version 9.0 (GraphPad Software, San Diego, CA, USA). This software was chosen for its advanced statistical capabilities and high-quality visualization features, ensuring clarity and precision in data presentation.

## 3. Results and Discussion

### 3.1. Characterization of the Protein Hydrolysates

**Figure 1:**
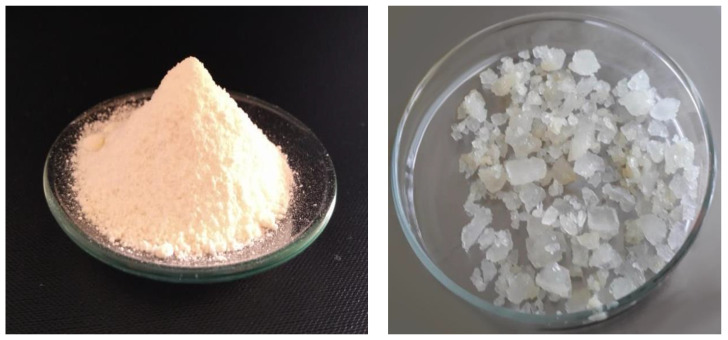
Protein hydrolysate.

The production of fish protein hydrolysates, available in both powdered and crystalline forms (Figure 1), has drawn increasing interest due to their potential to enhance food stability while offering health benefits. In this study, protein hydrolysates obtained from *Dosidicus gigas* through fermentation with Bacillus subtilis were analyzed for their bioactivity, focusing on antioxidant capacity and enzymatic inhibition related to chronic metabolic disorders.

#### 3.1.1. Antioxidant Capacity

Statistical analysis revealed significant differences (*p* < 0.05) in antioxidant activity and enzymatic inhibition among the hydrolysates (Table 1). For instance, hydrolysates with higher collagen content (H2 and H5) demonstrated significantly higher ORAC values (742.17 ± 12.45 μmol TE/g) compared to muscle-only formulations (H3), which showed lower activity (512.34 ± 10.87 μmol TE/g, *p* = 0.03). Homogeneity of variance was assessed using Levene’s test, confirming that the data met the assumptions for ANOVA (*p* < 0.05). Tukey’s post hoc test was employed to identify specific pairwise differences among the treatments.

The ORAC assay, a robust method to measure the ability to scavenge peroxyl radicals, revealed antioxidant activities ranging from 215.44 to 742.17 μmol TE/g sample. These values are comparable to hydrolysates obtained from salmon frames using alcalase and papain (544.25–840.03 μmol TE/g sample [35]). The high antioxidant capacity observed suggests that the hydrolysates might effectively counter oxidative stress in food matrices, prolonging shelf life and improving nutritional quality [36].

#### 3.1.2. Enzymatic Inhibition

Carbohydrate digestion was significantly modulated by the hydrolysates, which inhibited α-amylase (9.7–20.87%) and α-glucosidase (44.84–93.29%). The α-glucosidase inhibitory effect is comparable to acarbose, a clinical control, which showed 76.37% inhibition for this enzyme, while α-amylase retained approximately half of the enzymatic activity. The hydrolysates demonstrated potential to manage postprandial hyperglycemia, aligning with previous findings for crucian carp hydrolysates (11.36–20.83% [37]).

In terms of lipid metabolism, pancreatic lipase inhibition ranged from 11.16% to 35.44%, while Orlistat (1 mg/mL), a standard inhibitor, achieved 95.55%. These results suggest that, despite the moderate inhibition levels compared to Orlistat, this compound shows potential as an adjuvant in functional foods aimed at obesity management, particularly in supporting lipid metabolism regulation [38].

Furthermore, the hydrolysates exhibited angiotensin-converting enzyme (ACE-I) inhibition ranging from 22.11% to 88.96%, close to the inhibition observed with Captopril (87.23%). These findings indicate their potential as adjuvants for hypertension management, consistent with reports on hydrolysates from mackerel (60.4–77.9% [27]).

The bioactivity of these hydrolysates can be attributed to their peptide composition, which interacts with enzyme active sites and cofactors or induces conformational changes that impair enzymatic function; additionally, *Bacillus subtilis* fermentation enhances these properties through the production of specific metabolites [39]. This highlights the importance of considering the food matrix when incorporating these hydrolysates, as the interactions between peptides and other food components may influence their functionality.

#### 3.1.3. Applications in Functional Foods

The results underscore the potential of Dosidicus gigas hydrolysates as functional ingredients for nutraceuticals and food systems. Their ability to inhibit key enzymes and scavenge free radicals positions them as valuable candidates for clean-label formulations, aligning with consumer demand for health-promoting and sustainable food products.

### 3.2. Infrared Spectroscopy

**Figure 2:**
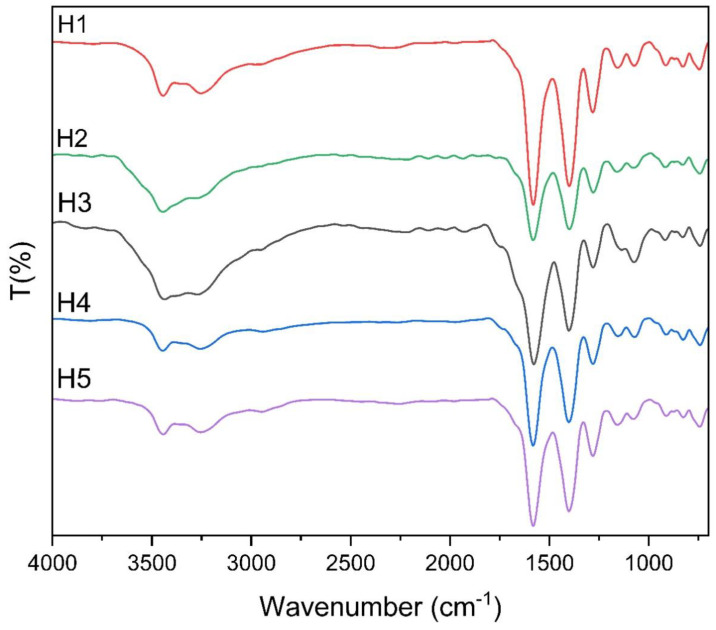
IR spectrum of lyophilized hydrolysates obtained from *Dosidicus gigas* protein by fermentation with *Bacillus subtilis*: (H1) 4 h of fermentation, 25% collagen, 75% muscle; (H2) 4 h of fermentation, 100% collagen; (H3) 8 h of fermentation, 100% muscle; (H4) 8 h of fermentation, 50% collagen, 50% muscle; (H5) 8 h of fermentation, 100% collagen.

Infrared spectroscopy was used to analyze the structural and functional groups present in the lyophilized hydrolysates derived from *Dosidicus gigas* proteins fermented with *Bacillus subtilis*. The results (Figure 2) revealed several characteristic absorption bands, providing insights into the molecular composition and bioactive potential of the hydrolysates [40].

Key absorption bands were identified at 1510 cm^−1^, 1392 cm^−1^, and 1198 cm^−1^, which align with findings reported by Verma et al. [41]. These bands are associated with the presence of biosurfactants produced during fermentation by *Bacillus subtilis.* Such surfactants are known for their amphiphilic nature, which enhances the solubility and functional properties of bioactive compounds in complex food systems.

The spectra also showed prominent bands at 3262 cm^−1^, corresponding to O-H stretching and bending vibrations, indicative of hydroxyl groups [13,42]. This suggests the presence of peptides or amino acids with hydroxyl-containing side chains, which are known to contribute to antioxidant activity by donating hydrogen atoms to neutralize free radicals.

Additionally, a strong absorption band at 1650 cm^−1^ was observed, representing C=O stretching vibrations from amide groups. This is characteristic of peptide bonds, confirming the hydrolyzed protein structure of the samples [13]. Aromatic rings were identified through C=C bending vibrations at 1582 cm^−1^, as well as aromatic C-H bending vibrations at 756 cm^−1^ and 856 cm^−1^ [13,43]. These features suggest the presence of aromatic amino acids, such as phenylalanine, tyrosine, or tryptophan, which are commonly associated with bioactivity in protein hydrolysates.

The FTIR analysis revealed the presence of key functional groups, such as hydroxyl (-OH), carbonyl (C=O), and aromatic rings, which are essential for the hydrolysates’ classification as biopolymers. These groups contribute directly to their bioactivity and functionality within food matrices. Specifically, hydroxyl groups play a critical role in redox reactions, enhancing antioxidant activity [44], while carbonyl groups facilitate interactions with matrix components, stabilizing emulsions and improving water-holding capacity [45]. Additionally, the amphiphilic nature of the hydrolysates, influenced by these functional groups, supports their dual role as bioactive compounds and structural stabilizers. This multifunctionality makes them highly effective for enhancing the physicochemical stability of food products, such as squid-based sausages, while also extending shelf life [46]. These findings align with the principles of polymer science, showcasing the potential of marine-derived biopolymers to act as natural alternatives to synthetic additives in food stabilization and preservation [18,47,48,49,50,51].

The FTIR analysis confirmed the presence of compounds characteristic of biosurfactants produced by *B. subtilis*, evidenced by peaks at 1510 cm^−1^, 1392 cm^−1^, and 1198 cm^−1^, which are associated with amphiphilic structures capable of inducing the formation of a distinct oily phase. Additionally, the spectra exhibited signals at 1582 cm^−1^, 856 cm^−1^, and 756 cm^−1^, corresponding to the presence of aromatic rings, suggesting the accumulation of metabolites with functional properties in the oily phase.

These findings indicate that the oily phase resulting from fermentation has a distinct chemical composition compared to the initial substrate and is free from substrate residue interferences, such as protein fragments and cells. This phenomenon is attributed to the action of enzymes and metabolites produced by *B. subtilis*, which modify the chemical structure of the medium and promote phase separation. Furthermore, this differentiation is not observed in the precipitation of the unfermented substrate, confirming that microbial activity plays a crucial role in generating an oily phase with distinctive properties [52,53,54].

The structural properties of the hydrolysates, as revealed by FTIR analysis, highlight their biopolymeric nature [55]. Peaks associated with hydroxyl groups (3262 cm^−1^), carbonyl groups (1650 cm^−1^), and aromatic rings (1510 cm^−1^) indicate the presence of functional peptide sequences [56]. These structural features play a crucial role in their functionality within food systems [57]. For example, hydroxyl and carbonyl groups contribute to the hydrolysates’ antioxidant capacity by participating in redox reactions, while aromatic rings enhance hydrophobic interactions within emulsions [58].

While FTIR allowed the identification of biosurfactants in the oily phase, complementary techniques are recommended to quantify these compounds and validate their concentration in fermented samples

FTIR analysis of the hydrolysates reveals the presence of key functional groups, including hydroxyl (-OH), carbonyl (C=O), and aromatic rings, which significantly influence their bioactivity and role in food matrix stability [40]. Specifically, hydroxyl groups contribute to redox reactions, enhancing antioxidant activity; carbonyl groups facilitate matrix interactions; and aromatic rings strengthen hydrophobic interactions, essential for emulsion stability.

Together, these structural properties support the hydrolysates’ multifunctionality, particularly in promoting antioxidant effects, stabilizing emulsions, and extending shelf life. Their potential as sustainable, clean-label additives aligns with industry trends toward natural food formulations [18,47,50,59].

### 3.3. Physicochemical Properties of the Sausage

#### 3.3.1. Proximal Analysis and Microbiological Quality

**Figure 3:**
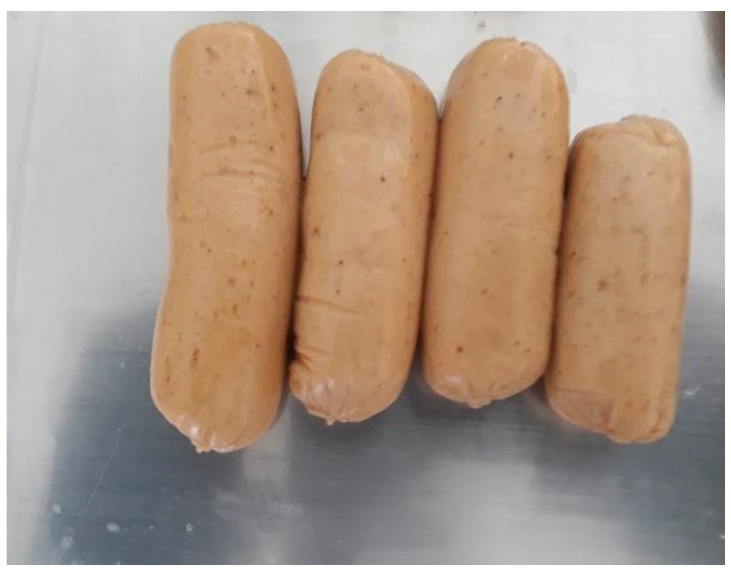
Jumbo squid sausage.

The jumbo squid sausage (Figure 3) was characterized by its proximal composition, yielding 65.48 ± 0.91% moisture, 16.87 ± 0.56% protein, 6.56 ± 0.22% lipids, 3.57 ± 0.33% fiber, 1.34 ± 0.26% ash, and 6.18% carbohydrates (calculated by difference). These values are consistent with the nutritional profile of squid-based sausages but show variations when compared to products formulated with different fat sources or protein proportions [60,61].

For instance, Feng & Arai [62] reported similar moisture content (64.07%) but observed lower protein (14.53%) and higher fat content (13.39%) in squid sausages enriched with lard. The differences can be attributed to the formulation used in the present study, which incorporates a higher proportion of squid muscle and reduced fat levels, resulting in a healthier nutritional profile. The high protein and reduced fat contents of the jumbo squid sausage may be particularly appealing to consumers seeking protein-rich, low-fat dietary options.

Microbiological analysis revealed the presence of coliforms at levels below 3 MPN/g (Most Probable Number per gram), while *Staphylococcus aureus* and *Salmonella* spp. were absent in 25 g of the product. These results indicate adequate hygienic handling and processing conditions, meeting food safety standards [63]. The absence of pathogenic microorganisms, combined with the low coliform counts, underscores the product microbiological stability and its suitability for consumption.

The determination of indicator microorganisms plays a crucial role in assessing the hygienic quality of food products during storage, particularly perishable items such as seafood-based sausages [62,64]. The low microbial counts observed in the present study suggest that the inclusion of functional hydrolysates and the processing methods employed may contribute to inhibiting microbial growth, aligning with previous findings on the antimicrobial properties of protein hydrolysates [11,65].

This highlights the potential of the hydrolysates not only as bioactive compounds but also as natural preservatives that enhance product safety and shelf life.

Overall, the proximal composition and microbiological quality of the jumbo squid sausage validate its nutritional and hygienic value. These findings support its potential application as a functional food, providing essential nutrients while maintaining food safety standards. Future studies could explore the impact of alternative formulations and storage conditions on the nutritional and microbiological stability of the product.

#### 3.3.2. Physicochemical Properties of the Sausages During Storage

The physicochemical properties (Table 2) of jumbo squid sausages with and without hydrolysates were monitored during 43 days of storage at 4 °C. Key parameters, including pH, total volatile bases (TVB), water-holding capacity (WHC), and color, were analyzed to assess the impact of hydrolysates on product stability.

##### pH

Throughout food processing, pH regulation is a determining factor in numerous biochemical and physicochemical processes. It directly impacts protein functionality, including denaturation and gelation, as well as enzymatic activity, microbial growth and lethality, bacterial spore activation or inactivation, and essential chemical reactions, such as the Maillard reaction [66]. A stable pH is crucial for preserving structural integrity, sensory attributes, and overall quality of food products.

As shown in Table 2, no significant differences were observed in the pH values between days 1 and 22 for both samples. However, by day 43, the sausage with hydrolysate exhibited a significant increase in pH (*p* < 0.05) from 6.51 ± 0.03 to 6.63 ± 0.07, likely due to protein deamination in meat emulsions and the microbial production of metabolites such as dimethylamine and trimethylamine [29]. The delayed pH increase in the hydrolysate-enriched sample suggests that microbial growth was effectively controlled, aligning with the antimicrobial properties of protein hydrolysates reported by Idowu et al. [35].

##### Total Volatile Bases (TVB)

Total volatile bases (TVBs) have long been employed as biochemical markers to evaluate fish quality and spoilage progression. However, the correlation between TVB accumulation and spoilage varies among fish species, and in some instances, TVB concentrations may not correspond with sensory assessments of degradation. To accurately define spoilage thresholds, further species-specific investigations are required to determine the TVB levels indicative of fish deterioration [67].

The results indicated that TVB values progressively increased in both groups, which is consistent with the natural degradation of proteins and microbial activity in marine meat products during refrigerated storage. However, sausages enriched with hydrolysates showed significantly lower TVB levels compared to the control group from day 22 onwards (*p* < 0.05). Specifically, at the end of the storage period, the hydrolysate sample presented 57.687 mg N/100 g, while the control sample reached 52.217 mg N/100 g. These values exceed the generally accepted limit for TVB-N in seafood products, which is 30 mg N/100 g, indicating that even with hydrolysates, spoilage processes were still occurring but at a slower rate compared to the control [68]. Based on these values, the sausages in this study exceeded the acceptability threshold between days 22 and 43, indicating that consumption beyond this period may not be recommended.

The reduction in TVB levels in treated samples is attributed to the antimicrobial properties of protein hydrolysates, which can inhibit spoilage microorganisms such as *Shewanella putrefaciens* and *Pseudomonas* spp., responsible for TVB generation in seafood products [69]. Additionally, bioactive peptides present in hydrolysates disrupt bacterial membranes, reducing their proliferation and the production of metabolites like trimethylamine and ammonia [65]. These findings align with previous research on protein hydrolysates in marine meat products, as Hajfathalian et al. [32] reported that adding fish roe hydrolysates to silver carp sausages resulted in a 30% reduction in TVB compared to untreated controls. Similarly, bioactive peptides in meat products act as enzymatic inhibitors, slowing proteolysis and the formation of volatile compounds associated with decomposition [31].

##### Water-Holding Capacity (WHC)

The ability of myofibrillar proteins to form gels plays a fundamental role in defining the physicochemical characteristics of meat products. During heating, these proteins create a three-dimensional matrix that entraps fat globules and significantly improves the water-holding capacity, contributing to the texture and stability of the final product [31].

No significant differences in WHC were observed during storage between the two samples. The presence of citrus fiber in the sausage formulation likely contributed to maintaining WHC due to its high content of hemicellulose, cellulose, and pectin. These components have excellent water-binding properties, which prevent liquid loss and enhance the structural integrity of the sausage [70].

##### Color

The CIELAB color space is defined by three coordinates: L*, representing lightness (100) to darkness (0); a*, spanning from negative values (green) to positive values (red); and b*, ranging from negative values (blue) to positive values (yellow), with both chromatic axes centered at 0 for a neutral gray [71].

Color changes during storage are summarized in Table 2. Luminosity (L*) decreased significantly (*p* < 0.05) over time, with the lowest values recorded for the hydrolysate-enriched sample on day 43 (H43). The redness (a*) increased significantly during storage (*p* < 0.05), with the highest values also observed for H43. No significant differences were found in yellowness (b*) between the samples on day 22, but b* values increased significantly by day 43 in both samples. These color changes can be primarily attributed to oxidative processes, which generate brown pigments as storage progresses, however, the antioxidant properties of hydrolysates likely slowed the rate of color deterioration, mitigating the impact of oxidative reactions [72]. The ability of hydrolysates to interact with lipid and aqueous phases suggests a potential role in stabilizing pigment molecules and reducing oxidative discoloration. Future studies should explore the synergy between hydrolysates and additional antioxidants to further enhance color stability in seafood-based emulsions [73].

The results obtained in this study highlight the biopolymeric nature of the hydrolysates derived from *Dosidicus gigas*, particularly their peptide-based structure, which includes functional groups such as hydroxyl, carbonyl, and aromatic side chains. These structural attributes enable the hydrolysates to act as multifunctional biopolymers in food matrices. Their amphiphilic properties facilitate interactions with both lipid and aqueous phases, improving emulsion stability and reducing phase separation [74]. Furthermore, the antioxidant and antimicrobial activities observed in this study are intrinsically linked to the hydrolysates’ peptide composition, which participates in redox reactions and disrupts microbial membranes. This dual functionality positions these hydrolysates as natural polymeric additives, offering structural stabilization while providing bioactive benefits. By leveraging these properties, the hydrolysates align with the principles of polymer science, addressing challenges in food stabilization and shelf-life extension. These findings underscore their potential as sustainable and clean-label alternatives to synthetic additives, contributing to the advancement of functional food systems.

##### Firmness Analysis

Firmness is a critical textural parameter influencing consumer acceptability. The analysis revealed no significant differences in firmness between the control (27.725 ± 1.51 N) and hydrolysate-treated samples (27.694 ± 1.98 N) throughout storage. This indicates that the incorporation of hydrolysates did not alter the structural integrity of the sausage matrix.

The presence of biopolymeric structures within the hydrolysates, including hydroxyl, carbonyl, and aromatic groups, may have contributed to maintaining the mechanical stability of the emulsified matrix, reinforcing the compatibility of hydrolysates with processed seafood formulations [32,46]. These findings suggest that hydrolysates can be integrated into formulations without negatively impacting textural attributes, further supporting their applicability in functional food development.

### 3.4. Microbiological Analysis

**Figure 4:**
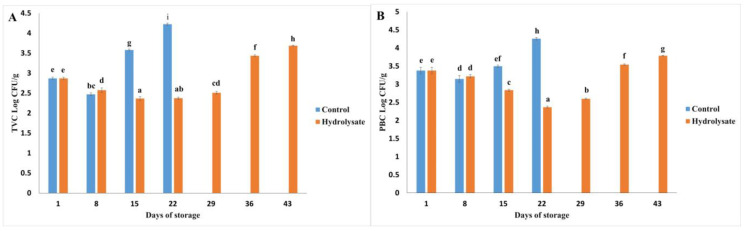
(**A**) Total viable count (TVC) and (**B**) psychrophilic bacteria count (PBC) of control sausages and sausages with hydrolysate in refrigerated storage. Bars indicate standard deviation (n = 3). Different letters indicate significative differences (*p* < 0.05).

The microbiological analysis of the samples, both with and without hydrolysates, revealed significant variations in microbial counts during the 43-day storage period at 4 °C. Total viable counts (TVCs) and psychrotrophic bacterial counts (PBCs) are illustrated in Figure 4, demonstrating the inhibitory effects of hydrolysates on microbial growth.

#### 3.4.1. Microbial Growth Trends

A significant increase in microbial counts was observed over time (*p* < 0.05). A temporary decrease in both TVC and PBC was noted on day 8 for all samples. This phenomenon may result from microbial antagonism, where the presence of spoilage bacteria inhibits the growth of competing species, as reported by Odeyemi et al. [69].

From day 15 onwards, the control samples exhibited a steady increase in microbial counts, indicating progressive spoilage. In contrast, samples enriched with hydrolysates showed a delayed microbial proliferation, with significantly lower counts maintained until day 22 (*p* < 0.05). These findings are consistent with the inhibitory effects of bioactive peptides derived from hydrolysates, which interact with bacterial cell membranes. Such interactions disrupt the lipid bilayer, induce phase separation, and cause membrane solubilization, thereby impairing bacterial viability [32].

#### 3.4.2. End of Storage Period

By day 29, a significant increase in microbial counts was observed in all samples. However, even at the end of the 43-day storage period, the microbial counts in hydrolysate-enriched samples remained below the acceptable limits for seafood products, as established by Hajfathalian et al. [32] for silver carp sausages (5 Log CFU/g, 30 days). Specifically, the final TVC and PBC values for hydrolysate-treated samples were 3.68 Log CFU/g and 3.78 Log CFU/g, respectively, highlighting the effectiveness of hydrolysates in extending product shelf life.

#### 3.4.3. Coliform Analysis

The absence of coliform bacteria throughout the storage period further underscores the microbiological stability of the samples. This indicates proper hygienic handling during production and the potential of hydrolysates as natural preservatives to mitigate microbial contamination [63,75,76].

The antimicrobial activity of hydrolysates can be attributed to the presence of small peptides with membrane-active properties. These peptides interfere with bacterial homeostasis by forming transient pores or channels within the membrane, disrupting nutrient transport and energy metabolism [32]. Additionally, hydrolysates may induce oxidative stress in bacterial cells, further compromising their survival [77].

## 4. Conclusions

This study demonstrated the significant bioactive potential of protein hydrolysates derived from the mantle of *Dosidicus gigas* through fermentation with *Bacillus subtilis*. The hydrolysates exhibited antioxidant activity by disrupting peroxyl radical chain reactions and effectively inhibiting key metabolic enzymes, including α-amylase, α-glucosidase, lipase, and angiotensin-converting enzyme I (ACE-I). These findings underscore their potential as functional ingredients for the management of metabolic disorders such as diabetes, obesity, and hypertension.

The integration of hydrolysates into jumbo squid sausages not only enhanced their nutritional profile—with higher protein and lower fat content compared to similar products reported in the literature—but also significantly improved food stability, extending their shelf life from 22 to 43 days. This effect was attributed to the hydrolysates’ ability to modulate microbial growth, reduce metabolite production, and delay oxidative browning, thereby enhancing the physicochemical stability of the product. These results support the application of *D. gigas* hydrolysates as natural stabilizers and clean-label additives, aligning with the increasing consumer demand for sustainable, health-promoting food products free from synthetic preservatives.

Furthermore, the biopolymeric nature of the hydrolysates allows their effective interaction with both lipid and aqueous phases, improving emulsion stability and contributing to food preservation. Their capacity to decrease total volatile bases (TVBs) and mitigate oxidative degradation presents a valuable alternative to conventional preservatives, offering a scalable and sustainable approach to enhancing the quality and safety of perishable food products.

From an industrial perspective, the utilization of *D. gigas* hydrolysates not only enhances product stability but also contributes to a circular economy model by valorizing marine by-products and reducing food waste. Future research should explore the synergistic effects of hydrolysates with other bioactive compounds, evaluate their impact on sensory properties in various food matrices, and assess their scalability for large-scale food production. These findings establish a solid foundation for the integration of marine-derived biopolymers into functional food systems, driving innovation in sustainable and health-oriented food technologies.

## Figures and Tables

**Table 1:** Characterization of *Dosidicus gigas* protein hydrolysates obtained via fermentation with *Bacillus subtilis* ATCC 6633.

	µmol Trolox Eq/g	Inhibition (%)	Inhibition (%)	Inhibition (%)	Inhibition (%)
Hydrolysate	ORAC	α-Amylase	α-Glucosidase	Lypase	Angiotensin-Converting Enzyme (ACE-I)
H1	337.70 ^c^	12.69 ^b^	78.52 ^c^	25.46 ^c^	62.2 ^c^
H2	383.59 ^d^	16.17 ^c^	80.76 ^c^	34.68 ^d^	71.85 ^d^
H3	215.44 ^a^	9.7 ^a^	44.84 ^a^	11.16 ^a^	22.11 ^a^
H4	742.17 ^e^	20.87 ^d^	93.29 ^d^	35.44 ^d^	88.96 ^e^
H5	266.88 ^b^	13.83 ^b^	62.42 ^b^	19.38 ^b^	48.73 ^b^

Different superscript letters within a column indicate significant differences between hydrolysates (*p* < 0.05).

**Table 2:** Physicochemical properties of jumbo squid sausages at the beginning (B) and after storage for 22 and 43 days with (H) or without (C) hydrolysates.

Sample	pH	TVB (mg N/100 g)	WHC (%)	L*	a*	b*
B	6.53 ± 0.05 ^ab^	2.387 ± 0.06 ^a^	86.08 ± 0.89 ^a^	74.32 ± 0.04 ^d^	7.13 ± 0.02 ^a^	34.51 ± 0.01 ^a^
C22	6.47 ± 0.05 ^a^	52.217 ± 0.65 ^c^	87.15 ± 0.96 ^a^	72.80 ± 0.02 ^a^	7.70 ± 0.02 ^c^	35.12 ± 0.02 ^b^
H22	6.51 ± 0.03 ^a^	33.578 ± 1.11 ^b^	87.46 ± 1.46 ^a^	73.17 ± 0.02 ^c^	7.50 ± 0.01 ^b^	34.59 ± 0.06 ^a^
H43	6.63 ± 0.07 ^b^	57.687 ± 2.4 ^d^	88.21 ± 0.36 ^a^	72.98 ± 0.05 ^b^	7.9 ± 0.03 ^d^	35.27 ± 0.03 ^c^

Results presented as Mean ± SD. Different superscript letters within a column indicate significant differences between sausages (*p* < 0.05).

## Data Availability

The original contributions presented in this study are included in the article. Further inquiries can be directed to the corresponding authors.

## Abbreviations

The following abbreviations are used in this manuscript:

MDPI	Multidisciplinary Digital Publishing Institute
ACE	Angiotensin-converting enzyme inhibition assay
ACE-I	Angiotensin-converting enzyme
AOAC	Association of Official Analytical Chemists
FTIR	Fourier Transform Infrared
GRAS	Generally recognized as safe
H_0_	A non-fermented control consisting of unprocessed muscle and collagen was included to assess baseline properties
H1	Sample conditions: 4 h of fermentation, 25% collagen, 75% muscle
H2	Sample conditions: 4 h of fermentation, 100% collagen
H3	Sample conditions: 8 h of fermentation, 100% muscle
H4	Sample conditions: 8 h of fermentation, 50% collagen, 50% muscle
H5	Sample conditions: 8 h of fermentation, 100% collagen
ORAC	Oxygen Radical Absorbance Capacity
MPN	Most Probable Number
PBCs	Psychrotrophic bacterial counts
TVBs	Total volatile bases
TVC	Total viable count
WHC	Water-holding capacity
